# Documentation and Testing of Illicit Drug Use on Inpatient Wards

**DOI:** 10.1192/bjo.2026.11813

**Published:** 2026-06-30

**Authors:** Ejura Opaluwah

**Affiliations:** Leeds and York Partnership Foundation Trust, Leeds, United Kingdom

## Abstract

**Aims:**

Illicit substance use contributes significantly to psychiatric morbidity and can complicate assessment, diagnosis, and treatment planning. NICE Guideline CG51 recommends that all adults presenting to mental health services receive a comprehensive assessment of substance misuse, including detailed historytaking, appropriate drug screening, and referral to addiction services when indicated. This clinical audit aimed to evaluate adherence to these standards within inpatient wards at the Becklin Centre. Specifically, it examined the documentation of illicit drug use, the level of detail provided, the use and recording of urine drug screening (UDS), and the provision of information and referrals to support services.

**Methods:**

A retrospective audit of 40 service users admitted for more than two weeks across three inpatient wards was conducted. Data were collected using a purposedesigned audit tool developed by the Clinical Effectiveness Team. Admission notes, medical entries, nursing documentation, and relevant forms from the first two weeks of admission were reviewed. Quantitative and qualitative data were analysed using Microsoft Excel. Thirteen patients without any history of illicit drug use were excluded from the detailed assessment, leaving 27 patients with documented histories of substance use.

**Results:**

Documentation of illicit drug use was present in 92% of all cases, indicating strong clinical awareness of substance misuse as part of psychiatric assessment. However, among patients with known substance use, only 67% had detailed histories describing specific substances, quantity, or frequency. Although UDS was clinically indicated for 70% of this group, only 29% underwent screening, with inconsistent documentation regarding reasons for omission. Test types and results were clearly recorded for only five patients.

Support pathways also demonstrated variability. While 67% of relevant patients were informed about addiction services, referrals were completed in only 14%, and documentation of patient refusal or clinical rationale was often absent. These findings highlight gaps in translating identification of substance use into structured assessment and onward referral. Contributing factors may include time pressures, acuity on admission, and poor knowledge of referral pathways.

**Conclusion:**

The audit shows that while initial enquiry regarding substance use is routinely performed, significant inconsistencies remain in detailed documentation, use of UDS, and referral to addiction services. To improve practice, interventions include visual reminders for staff to routinely offer UDS with consent, strengthened education on referral pathways and a planned re-audit within 12 months to evaluate improvement and support sustained change.

